# Human PrimPol Discrimination against Dideoxynucleotides during Primer Synthesis

**DOI:** 10.3390/genes12101487

**Published:** 2021-09-24

**Authors:** Gustavo Carvalho, Alberto Díaz-Talavera, Patricia A. Calvo, Luis Blanco, María I. Martínez-Jiménez

**Affiliations:** Centro de Biología Molecular “Severo Ochoa” (CSIC-UAM), c/Nicolás Cabrera 1, Cantoblanco, 28049 Madrid, Spain; gustavo.carvalho@umu.se (G.C.); adiazt@ext.cnio.es (A.D.-T.); pcalvo@cbm.csic.es (P.A.C.); lblanco@cbm.csic.es (L.B.)

**Keywords:** PrimPol, DNA primase, polymerase, dideoxynucleotides, CTNA, NRTIs, anti-retroviral, ddC, zalcitabine

## Abstract

PrimPol is required to re-prime DNA replication at both nucleus and mitochondria, thus facilitating fork progression during replicative stress. ddC is a chain-terminating nucleotide that has been widely used to block mitochondrial DNA replication because it is efficiently incorporated by the replicative polymerase Polγ. Here, we show that human PrimPol discriminates against dideoxynucleotides (ddNTP) when elongating a primer across 8oxoG lesions in the template, but also when starting *de novo* synthesis of DNA primers, and especially when selecting the 3′nucleotide of the initial dimer. PrimPol incorporates ddNTPs with a very low efficiency compared to dNTPs even in the presence of activating manganese ions, and only a 40-fold excess of ddNTP would significantly disturb PrimPol primase activity. This discrimination against ddNTPs prevents premature termination of the primers, warranting their use for elongation. The crystal structure of human PrimPol highlights Arg^291^ residue as responsible for the strong dNTP/ddNTP selectivity, since it interacts with the 3′-OH group of the incoming deoxynucleotide, absent in ddNTPs. Arg^291^, shown here to be critical for both primase and polymerase activities of human PrimPol, would contribute to the preferred binding of dNTPs *versus* ddNTPs at the 3′elongation site, thus avoiding synthesis of abortive primers.

## 1. Introduction

Chain-terminating nucleotide analogues (CTNAs) are typically characterized by substitutions on the 3′C sugar pentose of nucleotides by elimination of the 3′-OH group. A common class of CTNAs are 2′,3′-dideoxynucleotides (ddNTPs), which differ from their natural 2′-deoxynucleotides (dNTPs) counterparts in the lack of the 3′-OH group of the sugar. When a DNA polymerase incorporates a CTNA that cannot be proofread, the DNA synthesis is interrupted due to the lack of the 3′-OH group required for phosphodiester bond formation with the next incoming nucleotide. 

Nucleotide and nucleoside analogues have been widely and successfully used in antiviral therapy against HIV or hepatitis B as potent inhibitors of the viral reverse transcriptases (NRTIs). However, studies in patients have identified a series of adverse effects linking CTNA-based therapy to mitochondrial toxicity, mainly, via inhibition of mitochondrial Polγ [1,2]. In fact, mitochondrial Polγ was further classified as the most CTNA sensitive among the DNA polymerases found in human cells, because of its low discrimination between natural nucleotides and CTNAs [2]. According to this study, the selectivity range of NRTIs on polymerases is, in general: HIV-reverse transcriptase >> Polγ > Polβ > Pol**α**-primase = Polε; hence, it could be inferred that mitochondria is a primary cellular target for CTNA inhibition by mainly affecting mitochondrial DNA synthesis, and DNA repair to a lesser extent, since Polβ also localizes to the mitochondria [3,4]. Moreover, proofreading of incorporated CTNAs by Polγ happens very inefficiently, which results in their long persistence on the mitochondrial genome, thus contributing to the mitochondrial toxicity of these compounds [5]. Notably, among the first three nucleoside analogues approved by the FDA for anti-HIV/AIDS therapy (i.e., 3′-azido-2′,3′-dideoxythymidine, AZT; 2′,3′-dideoxycytidine, ddC; 2′,3′-dideoxyinosine, ddI), ddC is the most toxic for Polγ synthesis [2,5]. As ddC-induced mitochondrial toxicity is associated with severe adverse events (liver failure, pancreatitis, lactic acidosis, etc.), its commercialization as antiviral therapy has been discontinued in many countries. However, ddC has also been claimed to be an opportunity to poison cancer cells that relies heavily on mitochondrial function impairing mtDNA replication [6].

**Figure 1:**
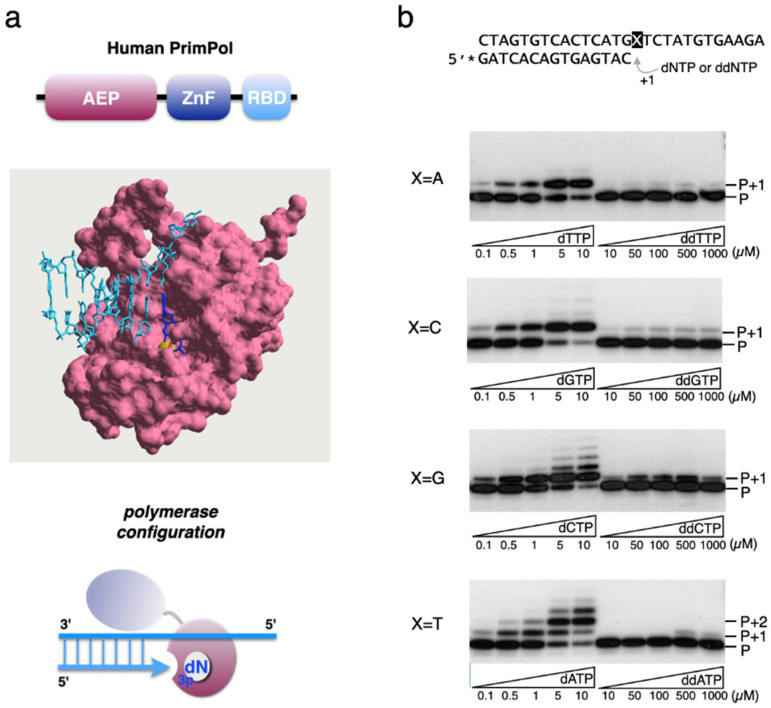
ddNTPs are not efficiently inserted during DNA polymerization by PrimPol. (**a**) Top: Schematic representation of the three domains of eukaryotic PrimPols: AEP (pink), Zn-finger domain (blue), and RPA-binding domain (cyan); centre: molecular surface representation of human PrimPol AEP core in pink (lacking the C-terminal region including the Zn-finger and RPA domains) forming a ternary complex with a DNA template/primer substrate (cyan), the incoming nucleotide (blue), and the metal cofactor (Ca^2+^ in yellow) were created with Swiss-PdbViewer (PDB id: 5L2X [18]); bottom: Schematics of PrimPol in polymerase configuration where the catalytic core (in pink) binds the 3′end of the primer (arrow in cyan) and the incoming deoxynucleotide triphosphate (in blue). C-terminal domain of PrimPol (in purple) does not collaborate in the binding. (**b**) PrimPol (200 nM) incorporation of individual dNTPs (0.1, 0.5, 1, 5 and 10 µM) or the equivalent ddNTP (10, 50, 100, 500 and 1000 µM) opposite each complementary template base (2.5 nM), and using Mn^2+^ as metal cofactor. P indicates the position of the 5′-labeled primer (indicated as *) that can be extended by one or more nucleotides (P + 1, P + 2...).

PrimPol is a DNA primase–polymerase, found both in nucleus and mitochondria of mammalian cells [7]. PrimPol contains an archeo-eukaryotic primase core (AEP) at the N-terminal, and a Zn finger domain followed by an RPA-binding domain at the C-terminal (Figure 1a). Two remarkable characteristics of PrimPol are its trans-lesion synthesis (TLS) ability to bypass 7,8-dihydro-8-oxoguanine (8oxoG) lesions [7,8,9], and its capacity to re-prime stalled forks in the nucleus [10], and also in the mitochondria [7,11], thus allowing an optimal fork elongation rate during DNA replication. Proliferation and survival of PrimPol-deficient cells was shown to be more sensitive to CTNAS, suggesting that PrimPol re-priming is beneficial when CTNAs stall the replication process [12]. Torregrosa-Muñumer et al. [13] analysed how mtDNA replication intermediates responded to a low dose of ultraviolet radiation, oxidative damage, or to the presence of ddC. As mentioned, ddNTPs are potent inhibitors of the mitochondrial replicase, DNA Polγ, as they are easily inserted but resistant to be proofread by this polymerase [2]. Unexpectedly, the presence of moderate concentrations of ddC altered the type of mtDNA replication intermediates, suggesting the participation of a primase activity [13]. In agreement with the localization of PrimPol in mitochondria, PrimPol-deficient cells were not able to produce these specific replication intermediates, allowing us to conclude that PrimPol helps mtDNA replication by reinitiating forks stalled by ddC-primer incorporation, and also by re-priming from nonconventional origins [11]. In concordance, a recent work reported a patient with mitochondrial toxicity associated with antiviral therapy against HIV who presented an inactivating mutation at the catalytic centre of PrimPol [14]. Therefore, PrimPol has a protective role in mitochondrial toxicity induced by NRTI antiviral therapy [14].

Previous studies [15] showed that the polymerase activity of human PrimPol is able to incorporate four of the eight NRTIs approved by the FDA from 40-fold to 1000-fold less efficiently than natural NTPs [15]. However, little is known about the effect of NRTIs on the primase activity of PrimPol, especially during the initial steps of primer synthesis, which likely require additional interactions with the incoming 3′nucleotide [16,17]. As shown here, catalysis of the initial dimer by human PrimPol is especially reluctant to incorporate a ddNTP, and Arg^291^ plays a crucial role in dNTP binding at the 3′elongation site, mediating a strong discrimination against ddNTPs, which minimizes synthesis of abortive primers.

## 2. Materials and Methods

### 2.1. Reagents and Oligonucleotides

Inorganic salts, acids, bases, and organic compounds were purchased from Merck (Kenilworth, NJ, USA), Sigma Aldrich (St Louis, MO, USA), and AppliChem (Darmstadt, Germany). Chromatography resins for protein purification used in this work were Ni-NTA Superflow purchased from Qiagen (CITY, Germany) and Heparin Sepharose Fast Flow to GE Healthcare (Fairfield, CT, USA). Ultrapure *NTPs*, dNTPs, and ddNTPs were supplied by GE healthcare (Fairfield, CT, USA). Labelled nucleotides and [α-^32^P]dGTP were purchased from Perkin Elmer (Waltham, MA, USA). T4 polynucleotide kinase for DNA labelling was obtained from New England Biolabs (Ipswich, MA, USA) and used as indicated by the manufacturer. Vent polymerase was supplied by New England Biolabs (Ipswich, MA, USA). DNA oligonucleotides were synthesized by Sigma Aldrich (St Louis, MO, USA) and purified by 8 M urea-20% polyacrylamide gel electrophoresis. Oligonucleotides containing 8-oxoG were purchased from Eurogentec (Seraing, Belgium). 

### 2.2. Human PrimPol and Mutants

Human PrimPol point mutations (R291A and R291K) were generated with the QuikChange Site-Directed Mutagenesis protocol from Stratagene (La Jolla, CA, USA) using as a template the expression vector pET16::CCDC111 [7] and the following primers: R291A-forward (5′ CAAGAAATAGAAACTTTGCGCTATATAAATCATCAAAAATTGG 3′); R291A-reverse (5′ CCAATTTTTGATGATTTATATAGCGCAAAGTTTCTATTTCTTG 3′); R291K-forward (5′ CAAGAAATAGAAACTTTAAGCTATATAAATCATCAAAAATTGG 3′); R291K-reverse (5′ CCAATTTTTGATGATTTATATAGCTTAAAGTTTCTATTTCTTG 3′). PrimPol point mutations were confirmed by sequencing. Overexpression and purification of both wild-type (WT) PrimPol, R291A, and R291K variants were carried out as previously described [7].

### 2.3. Polymerase Assay on Specific Primer:Template Molecules

A 5′ ^32^P-labelled primer (5′ GATCACAGTGAGTAC 3′) was hybridized to a complementary template (5′AGAAGTGTATCTXGTACTCACTGTGATC 3′ where X corresponds to the indicated nucleotide A/G/C or T) to evaluate the insertion of the corresponding complementary dNTP or ddNTP (Figure 1b). DNA polymerase assays in the presence of various concentrations of the four dNTPs were carried out using a 5′ ^32^P-labelled primer (5′ CTGCAGCTGATGCGCC 3′) hybridized to a complementary template (5′GTACCCGGGGATCCGTACGGGCGCATCAGCTGCAG 3′), as indicated in Figure 5e. All reaction mixtures (in 20 µL) contained Buffer R, 2.5 nM [γ-^32^P]-labelled primer:template, 200 nM purified WT PrimPol or its variants, and the indicated dNTPs or ddNTPs concentrations. After incubation during 30 min at 30 °C, reactions were stopped by adding 8 μL of formamide loading buffer, loaded onto 8 M urea-containing 20% polyacrylamide sequencing gels, and autoradiography was used to detect primer extension. The results shown in Figure 1b and Figure 5d are representative of at least three different experiments.

### 2.4. Steady-State Kinetics Assay

Kinetic parameters were obtained by measuring the formation of a +1 primer-extended product by PrimPol. DNA primer/template structure (250 nM) used as substrate was: 5′ CTGCAGCTGATGCGCC 3′/5′ GTACCCGGGGATCCGTACXGGCGCATCAGCTGCAG 3′ (where X = A, T, C, G or 8oxo-G). Reactions were carried out (in 20 µL) using Buffer R (50 mM Tris–HCl pH 7.5, 40 mM NaCl, 2.5% (*w/v*) glycerol, 1 mM DTT, 0.1 mg/mL BSA, 1 mM MnCl_2_), 40 nM purified PrimPol, and increasing concentrations of the complementary dNTP (0,25 µM to 10 µM for the WT, and 1 µM to 500 µM for the mutants) or ddNTP (2.5 µM to 350 µM for the WT). After short periods of incubation (5 min for the WT and R291K with dNTP and 15 min for the WT with ddNTP or R291A with dNTP) at 30 °C, reactions were stopped and the products resolved as previously described. A BAS reader 1500 (Fujifilm) was used to detect +1 primer-extended products, which were then quantified by densitometry using ImageJ software. The observed rate of nucleotide incorporation (+1 extended primer) was plotted as a function of nucleotide concentration. Data were fit to the Michaelis–Menten equation using non-linear regression to determine the apparent K_m_ and k_cat_ parameters. Two different experiments containing a wide range of dNTP concentrations were used to obtain the kinetic parameters.

### 2.5. Exonuclease and Pyrophosphorolysis Assay

**Figure 2:**
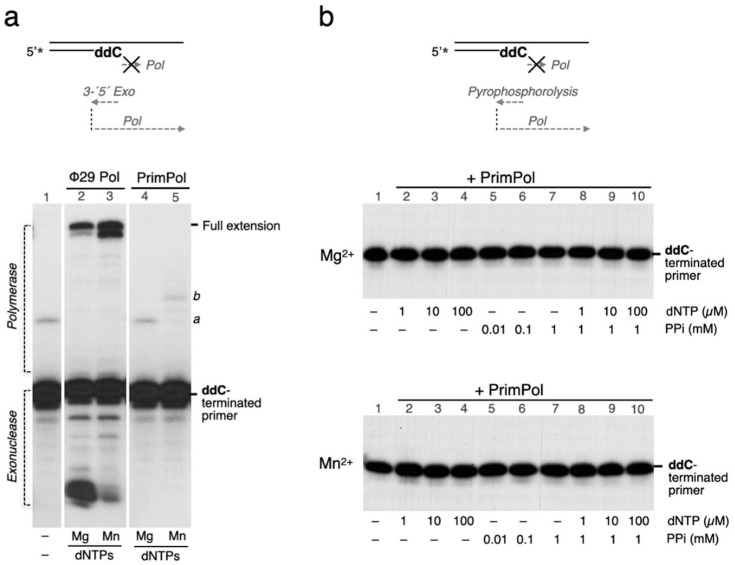
PrimPol cannot remove a 3′-terminal ddCMP. (**a**) Phi29-encoded DNA polymerase (50 nM) was used as a positive control to degrade (via 3′–5′ proofreading) a 2′,3′-dideoxynucleotide (ddC) present at the primer *terminus*, generating an unblocked primer terminus that can be fully extended in the presence of dNTPs (100 µM) and Mg^2+^ (5 mM) or Mn^2+^ (1mM) as metal cofactor (lanes 2 and 3). Conversely, PrimPol (50 nM) is not able to remove a 3′-terminal ddCMP with any of the two activating metals, thus impeding elongation of the ddC-terminated primer. *a*, labelled artefact, present in the original DNA (lane 1); *b*, dNTPs + Mn^2+^-driven extension of the artefact band (lane 5). (**b**) PrimPol (200 nM) is not able to remove a 2′,3′-dideoxynucleotide (ddC) present at the primer *terminus* by pyrophosphorolysis, neither with Mg^2+^ (upper panel) or Mn^2+^ (lower panel). Increasing concentration of PPi (0.01, 0.1, 1 mM), alone or in combination with dNTPs (1, 10, 100 µM), did not produce any ddC removal from the primer terminus or the subsequent elongation.

Using a 5′ ^32^P-labelled primer where the 3′ end of the primer is a chain-terminating ddCMP residue (5′ GGCGACGGCCAGTddC 3′) hybridized to a complementary template substrate (5′ CCGCTGCCGGTCAGTGACGATCGTGACTGC 3′), 3′ to 5′ exonuclease and pyrophosphorolysis activities were tested. Polymerization of dNTPs can only occur after degradation of the ddCMP residue. In the 3′–5′exonuclease/polymerization assay (Figure 2a), reactions in buffer R, containing 10 mM MgCl_2_ or 1 mM MnCl_2_ as a metal cofactor and 100 µM dNTPs, were incubated with 50 nM bacteriophage Phi29-encoded DNA polymerase as positive control or 200 nM PrimPol during 1 h at 30 °C. The pyrophosphorolysis/polymerization assay (Figure 2b) was carried out in the presence of human PrimPol (200 nM), the indicated concentration of dNTPs (1, 10, and 100 µM), or PPi (0.01, 0.1, and 1 mM) in buffer R with the mentioned metal cofactors and the primer/template described above. After 1 h at 30 °C, reactions were resolved and analysed as described for the polymerase reaction. The results shown in Figure 2 are representative of at least three different experiments 

### 2.6. Primase Assay on Specific Oligonucleotide Templates

**Figure 3:**
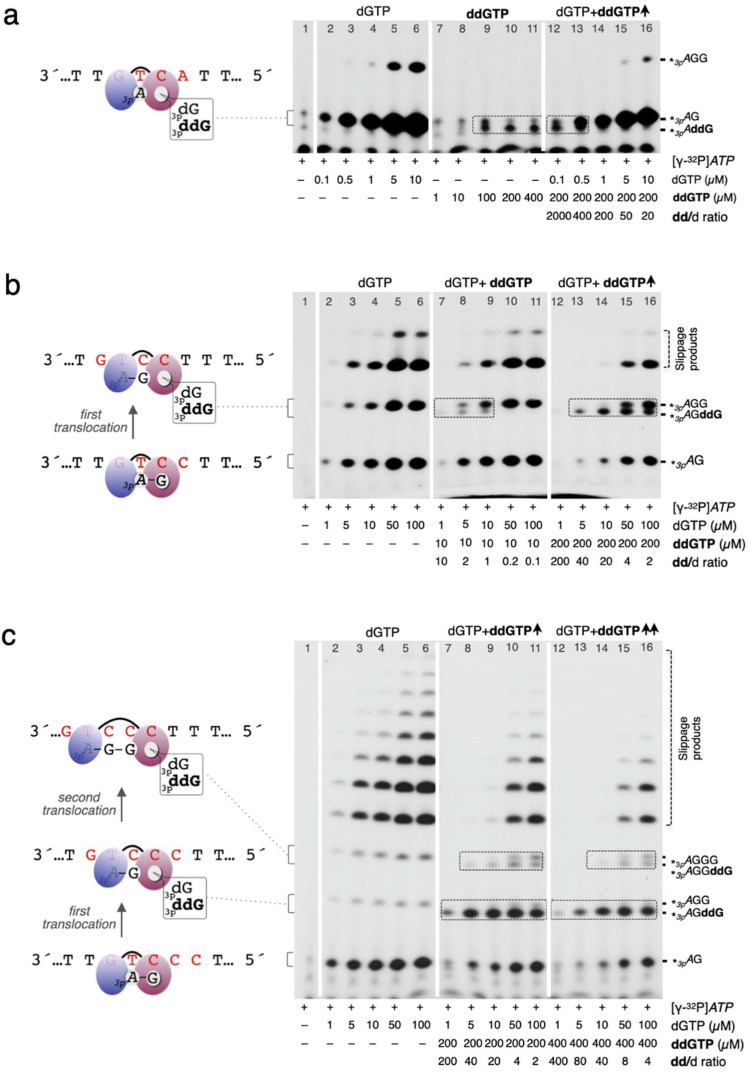
PrimPol shows a strong discrimination against ddNTPs during initiation of primer synthesis. (**a**) *Dimer* formation: PrimPol strongly discriminates against ddNTPs when making the initiating dimer. The left panel shows conventional *_3p_A*dG primer formation on the GTCA template. The middle panel shows how inefficient the reaction is when natural dGTP is substituted by ddGTP to form a dimer, where more than 100 µM ddGTP is needed to allow dimer formation. The right panel demonstrates that at least a 400-fold higher ddGTP concentration over dGTP is needed to detect *_3p_A*ddG. Note that products containing ddG at the 3′ end *terminus* run slightly faster than their dC-terminated counterparts. (**b**) *Trimer* formation: PrimPol poorly discriminates against ddGTP when catalysing a trimer. The left panel shows conventional *_3p_A*dGdG primer formation on the GTCC template. In the middle panel, dimers containing ddG (*_3p_A*ddG) are not detected when using a dGTP:ddGTP ratio of 1:200; conversely, ddG-terminated trimers (*_3p_A*dGddG) can be observed with a low dGTP/ddGTP ratio of 1:2 in the middle and right panels. (**c**) *Tetramer* formation: PrimPol improves discrimination against ddGTP during tetramer formation. By using a 3′ GTCCC 5′ template, which allows the synthesis of a canonical tetramer (*_3p_A*GGG) and abundant slippage products, trimers containing a 3′-terminal ddG (*_3p_A*dGddG) accumulate and some blocked tetramers are also produced by PrimPol (400 nM). Despite a global inhibition, most products can be extended when the ddGTP/dGTP ratio is below 8. Illustrations of each reaction appear at the left side of the gels. *_3p_A* represents the initiating nucleotide [γ-^32^P]*ATP* (provided at 16 nM), located at the 5′ primer site of PrimPol and complementary to the thymine of the 3′GTC priming site in the template). *_3p_*dG and *_3p_*ddG represent the alternative nucleotides (dGTP and ddGTP) to occupy the 3′ elongation site of PrimPol, paired to the cytosine of the 3′GTC priming site (**a**) or to be paired to other cytosine residues to make a trimer (**b**) or a tetramer (**c**). PrimPol was used at 400 nM, the template at 1 µM, and the indicated concentration of dGTP and ddGTP ddG-terminated primers and their corresponding dG-terminated primers (when present) are highlighted with a grey dashed square.

**Figure 4:**
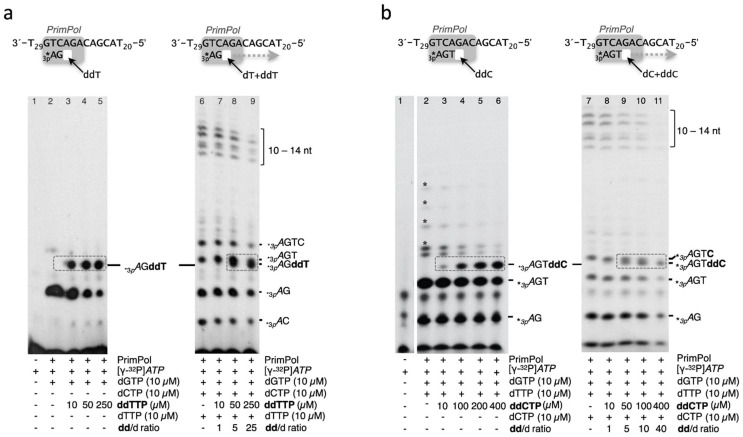
PrimPol shows a poor discrimination against ddNTPs, especially when making trimers. (**a**) Left panel shows *_3p_A*dGddT *trimers* formed by PrimPol (400 µM) when using a heteropolymeric template (see the scheme) at 1 µM, and providing [γ-^32^P]*ATP* (16 nM), dGTP (10 µM)*,* and ddTTP at a concentration as low as 10 µM; the right panel evidences that further addition of dCTP and the competing dTTP at 10 µM still allows the formation of *_3p_A*dGddT *trimers* when providing at least a 5-fold excess of ddTTP over dTTP, consequently reducing the amount of longer extension products (lane 8), which is more obvious when using a 25-fold excess of ddTTP (lane 9). (**b**) The left panel exhibits the abortive tetramers (*_3p_A*GTddC) synthesized by PrimPol when ddCTP (lanes 3–6) is added as a unique source of cytosine; the right panel demonstrates that at least a 5-fold excess of ddCTP over dCTP is required to observe the blocked tetramers, and inhibit further extension of the primers (lanes 9–11). ddNTP-terminated primers and their corresponding dNTP-terminated primers (when present) are highlighted with a grey dashed square.

**Figure 5:**
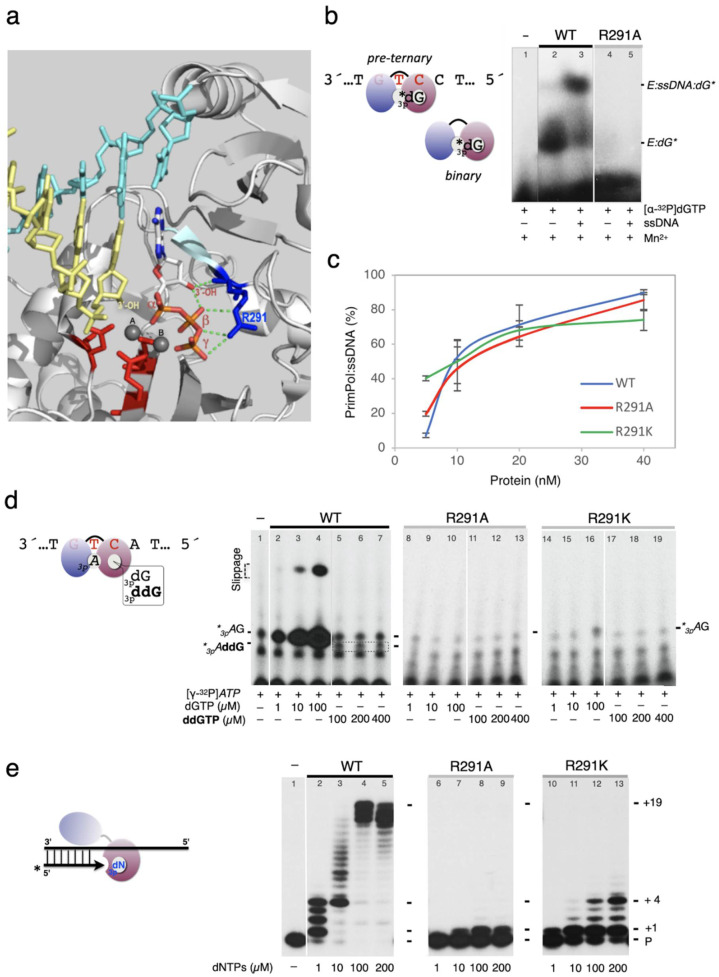
Critical role of Arg^291^, a ligand of the 3′-OH and the β-γ phosphate moieties of incoming 3′ dNTPs. (**a**) Ribbon representation of the ternary complex of *Hs*PrimPol:DNA:dATP, created with PyMol Molecular Graphics System (PDB id: 7JL8; [9]), depicting the interaction of PrimPol (grey) with DNA (primer strand is coloured in yellow and templating strand in cyan) and 3′ incoming dATP. Residues acting as metal ligands (Asp^114^, Glu^116^, Asp^280^) are indicated in red, and the two metal ions A and B (Ca^2+^) as grey spheres. The invariant Arg^291^ (R291; indicated in blue) interacts with the 3′-OH of dATP, forming a hydrogen bond with its backbone amino group, and also interacts with β and γ phosphates of the incoming nucleotide, likely contributing to the correct orientation and stability of the substrate, and catalysis of the phosphodiester bond. In addition, it could be responsible for the strong preference for dNTPs over ddNTPs. (**b**) PrimPol:dNTP (binary) and PrimPol:ssDNA:dNTP (ternary) complexes detected by EMSA, using [α-^32^P]dGTP (16 nM), 1 mM MnCl_2_ as a source of the required activating Mn^2+^ ions, and PrimPol WT or R291A variant (1 µM), either without (lane 2 and 4) or with (lanes 3 and 5) 3′T_36_GTCCT_20_5′ (500 nM) as an ssDNA template. Mutation of Arg^291^ to Ala completely abolished the capacity of PrimPol to form a binary complex (E:dGTP*) or pre-ternary complex (E:ssDNA:dGTP*) (lanes 4 and 5), which are pre-catalytic steps during primer synthesis. (**c**) Structural integrity of the R291K and R291A mutants was assessed by their wild-type capacity to bind ssDNA. The plot represents the percentage of PrimPol:ssDNA complex formed at increasing concentrations of each PrimPol, analysed by EMSA as described in the Materials and Methods. (**d**) Dimer formation assay on the GTCA template (1 µM), either with PrimPol WT or two Arg^291^ mutants (R291A and R292K) (400 nM), 1 mM MnCl_2_, γ-^32^P]*ATP* (16 nM) as the 5′ site nucleotide, and dGTP (1, 10, 100 µM) or ddGTP (100, 200, 400 µM) at the indicated concentrations as the 3′ site nucleotide (see the schematics). WT PrimPol makes dimers with dGTP (lanes 2–4), but very inefficiently with ddGTP (grey dashed box; lanes 5–7). Mutant R291K and R291A were inactive with dGTP or ddGTP (lanes 8–19). (**e**) DNA polymerase (primer elongation) assay on a pre-existing template/primer (2, 5 nM), either with PrimPol WT or mutants R291A and R292K (200 nM), 1 mM MnCl_2_ as metal cofactor, and increasing concentrations (1, 10, 100 µM) of the 4 dNTPs. The schematics emphasize our model for PrimPol elongation under these conditions, which requires mobilization of the Zn-finger domain (in blue). Unlike the wild-type *Hs*PrimPol, both mutants at Arg^291^ displayed a very limited (R291K) or null (R291A) primer elongation (DNA polymerase) activity, indicating that Arg^291^ is crucial for dNTP binding under these conditions.

Primase assays (Figure 3, Figure 4 and Figure 5d) were carried out using ssDNA oligonucleotide templates containing the preferred priming site GTC [7], flanked by thymines (3′ T_10_-GTCC-T_15_ 5′). Variations around this sequence are indicated in each experiment, including a more extended heteropolymeric region following the 3′GTC preferred priming site (3′ T_29_GTCAGACAGCAT_20_ 5′) used to study full elongation of the primers. The reaction mixture (20 µL) contained Buffer R, 1 µM ssDNA template, 400 nM PrimPol, 16 nM [γ-^32^P]*ATP,* or [α-^32^P]dGTP (250 µCi; 3000 Ci mmol^−^^1^) and *NTPs*, dNTPs, or ddNTPs at the concentration indicated in the figures. After incubation for 60 min at 30 °C, reactions were stopped by adding 8 μL of formamide loading buffer (95% formamide, 20 mM EDTA, 0.1% xylene–cyanol, and 0.1% bromophenol blue) and the products resolved by 8 M urea-containing 20% polyacrylamide sequencing gels. Following electrophoresis, de novo synthesized primers were detected by autoradiography and the images analysed with ImageJ software. The discrimination value (dNTP/ddNTP) to form dimers was calculated from the relative intensities of the *_3p_A*G versus *_3p_A*ddG bands, corrected by the concentration ratio of each 3′ nucleotide. The discrimination value (dNTP/ddNTP) to form a trimer was calculated considering the fraction of trimers ended with dNTP (+ further elongated products), divided by the amount of ddNTP-terminated trimer, corrected by the concentration ratio of each 3′ nucleotide provided. Similar quantitation was performed to determine the d/dd discrimination during tetramer formation. The primase assays shown in Figure 3, Figure 4 and Figure 5d are representative of at least three different experiments.

### 2.7. EMSA for PrimPol:dNTP Binary and PrimPol:ssDNA:dNTP Preternary Complex

Binary complex (PrimPol:[α-^32^P]dGTP) and pre-ternary complex (PrimPol:ssDNA:[α-^32^P]dGTP) formed with PrimPol (1 µM), [α-^32^P]dGTP (16 nM), 1 mM MnCl_2_, and ssDNA 3′ T_29_-GTCC-T_25_ 5′ (0.5 µM) when indicated, was evaluated in Buffer D (50 mM Tris–HCl pH 7.5, 40 mM NaCl, 2.5% (*w/v*) glycerol, 1 mM DTT, 0.1 mg/mL BSA). Reactions (20 µL final volume) were incubated for 10 min at 30 °C. After addition of 2 µL of loading Buffer S (50% glycerol, 0.1% (*w/v*) xylene–cyanol, and 0.1% (*w/v*) bromophenol blue), and binary and preternary complexes were analysed by EMSA in a Tris-glycine (pH 8.3) native 6% polyacrylamide gel, resolved at 180 V for 120 min at 4 °C, and vacuum-dried. The mobility shift of free [α-^32^P]dGTP *versus* PrimPol:[α-^32^P]dGTP and PrimPol:ssDNA:[α-^32^P]dGTP complexes was analysed by autoradiography. The EMSA assay shown in Figure 5b is representative of at least three different experiments.

### 2.8. EMSA for PrimPol:ssDNA Binary Complex

Binary complex (PrimPol: [γ-^32^P]-labelled ssDNA) formation was tested either with increasing concentrations (5–40 nM) of either wild-type PrimPol or mutants R291A and R291K and [γ-^32^P]-labelled ssDNA 3′ T_29_-GTCC-T_25_ 5′ (2 nM), in Buffer D (50 mM Tris–HCl pH 7.5, 40 mM NaCl, 2.5% (*w/v*) glycerol, 1 mM DTT, 0.1 mg/mL BSA and 2.5% PEG 4000). Reactions (20 µL final volume) were incubated for 10 min at 30 °C, and processed as described above for other EMSA assays. The mobility shift of the free [γ-^32^P]-labelled ssDNA band to form a PrimPol:ssDNA complex was analysed by autoradiography and quantitated by densitometry of the autoradiographs. The plot shown in Figure 5c corresponds to at least three different experiments.

## 3. Results

### 3.1. Human PrimPol Discriminates against ddNTPs during Polymerization and TLS

In addition to its unique ability as a DNA primase [7], PrimPol can use a polymerase configuration to extend a pre-existing primer (Figure 1a). To analyse the susceptibility of PrimPol to incorporate ddNTPs during polymerization in our experimental conditions, we measured the individual kinetic constants of each dNTP’s versus ddNTP’s incorporation (Table 1), providing a primer:template DNA substrate and manganese as the preferred metal activator (as indicated in the Materials and Methods). As expected, PrimPol displayed a strong discrimination against ddNTPs (Figure 1b), where even a 1000-fold higher ddNTP concentration does not allow a comparable +1 insertion relative to that of the cognate dNTP. A shown in Table 1, steady-state analysis of the insertion of each individual dNTP compared to its corresponding ddNTP allowed us to conclude that the catalytic efficiency ([k_cat_/K_m_]_d_/[k_cat_/K_m_]_dd_) of dNTPs was 325- to 719-fold higher than their ddNTP counterparts. Analysis of the individual kinetic parameters indicated that PrimPol has a lower affinity for ddNTPs (K_m_ is up to 72-fold higher for ddNTPs than for dNTPs) and a decreased catalytic constant between 1 and 2 orders of magnitude (Table 1). The discrimination between dATP and ddATP obtained from our study is in concordance with that reported previously [15]. However, we found a discrepancy of 10-fold in the relative incorporation efficiency of dCTP/ddCTP which could be explained by the sequence context of the DNA substrate used in each study.

Moreover, we analysed if human PrimPol efficiently discriminates against ddNTPs when nucleotide insertion occurs opposite 8oxoG, a highly frequent oxidative lesion whose bypass is a hallmark of PrimPol TLS activity [7,8]. As shown in Table 1, the calculated dCTP/ddCTP discrimination was 345, quite similar to the value obtained for insertions opposite an undamaged dG templating base (330; see Table 1). On the other hand, no insertion of ddATP opposite 8oxoG was observed, and dATP/ddATP discrimination could not be determined (Table 1), thus being higher than the discrimination value for dATP/ddATP insertions opposite an undamaged dT templating base (719; Table 1). These results allowed us to conclude that human PrimPol strongly discriminates against ddNTPs during polymerization and TLS.

### 3.2. Human PrimPol Cannot Remove Inserted Dideoxynucleotides 

The presence and permanence of ddNTPs on the DNA will depend primarily on the balance between the incorporation rate of these nucleotide analogues by DNA polymerases and the capacity to remove them either by the intrinsic 3′–5′ proofreading activity of some DNA polymerases, or by stand-alone nucleases. The first line of defence against a misincorporated nucleotide is the 3′–5′ exonuclease activity that is intrinsic to replicative DNA polymerases and responsible for the removal of mispaired nucleotides or nucleotide analogues that block elongation, such as CTNAs. Among the eukaryotic polymerases, only the replicative polymerases Polδ and Polε (nuclear) and Polγ (mitochondrial) are endowed with an active and evolutionarily conserved 3′–5′ proofreading domain [19,20]. As shown in Figure 2a (lanes 2–3), Phi29 DNA polymerase, which has a potent 3′–5′ exonuclease [21,22] was able to remove ddC from a ddC-terminated primer and to catalyse its full extension in the presence of dNTPs (lanes 2–3). In agreement with the lack of a proofreading domain, PrimPol could not remove ddC from the ddC-terminated primer, thus precluding primer extension when providing dNTPs (Figure 2a, lanes 4–5). Other polymerases devoid of 3′–5′exonucleolytic proofreading, such as Polλ, can take advantage of a polymerization reversal step triggered by pyrophosphate (i.e., pyrophosphorolysis) that can serve to edit a misincorporated nucleotide [23]. To evaluate if PrimPol has pyrophosphorolysis activity, the ddC-terminated primer/template was incubated with increasing concentrations of PPi (Figure 2b, lanes 5–7), in the presence of either magnesium ions (upper panel) or manganese ions (lower panel). Even at 1 mM PPi, no PPi-mediated excision of the terminating ddC was observed (lane 7). Moreover, addition of both dNTPs and PPi did not render any primer elongation (lanes 8–10), as observed in the absence of PPi (lanes 1–4). Therefore, PrimPol is not endowed with a mechanism able to eliminate an inserted dideoxynucleotide, neither by 3′–5′exonucleolysis nor by pyrophosphorolysis. It is likely that the presence of any of these activities would compromise the catalytic efficiency of PrimPol when catalysing rate-limiting steps associated with the synthesis of DNA primers. Thus, it was crucial to investigate if PrimPol has a strong discrimination against inserting ddNTPs during the first events leading to primer synthesis.

### 3.3. Human PrimPol Discriminates against ddNTPs When Synthesizing Primers

Incorporation of a CTNA (i.e., ddNTP) by Polγ will interrupt mtDNA replication, and the restart of DNA synthesis would require a re-priming event executed by PrimPol [11]. Therefore, it is relevant to demonstrate that PrimPol can synthesize mature primers in the presence of ddNTPs, specially assessing the discrimination against these CTNAs during the initial steps of primer synthesis; from dimer to tetramer, and its further extension to form a mature primer. For delineating each individual step, it is essential to analyse the primase activity of PrimPol in a single-sequence context. Accordingly, we provided a single-stranded (ssDNA) template oligonucleotide containing a favoured priming sequence 3′ GTC 5′ (and variations, as indicated) flanked by homopolymeric dT tails, as previously reported [7,16].

Firstly, we wanted to know if PrimPol discriminates against ddNTPs during dimer formation, which is the first catalytic step of primer synthesis. On the ssDNA template oligonucleotide containing the sequence 3′ GTCA 5′ (see the schematics in Figure 3a), PrimPol favours the use of ‘TC’ as a template to generate a *_3p_A*G dimer when providing 16 nM [γ-32P]ATP as the initiating 5′-ribonucleotide, and dGTP (or ddGTP) as the incoming 3′ nucleotide, in the presence of activating manganese ions [16]. As shown in Figure 3a (lanes 2–6), PrimPol synthesized *_3p_A*G dimers very efficiently, even when dGTP was provided at a concentration as low as 0.1 μM. On the contrary, some *_3p_A*ddG dimers were only seen when ddGTP was supplied at 100 μM or higher concentrations (Figure 3a, lane 9–11); moreover, increasing concentrations of ddGTP did not result in accumulation of more *_3p_A*ddG dimers, indicating a catalytic defect, and suggesting a large preference of PrimPol for dNTPs during initiation of primer synthesis. When both dGTP and ddGTP were supplied in the same reaction, *_3p_A*ddG dimers were more abundant than *_3p_A*G dimers only when the ddGTP/dGTP ratio was higher than 2000-fold (Figure 3a, lane 12). Otherwise, just a slight increase in dGTP concentration resulted in the generation of *_3p_A*G as the main dimers synthesized by PrimPol (Figure 3a, lane 13–16). The quantification of the dNTP/ddNTP discrimination to form a dimer, calculated as indicated in the Materials and Methods, was 1400-fold.

Next, we used an ssDNA template with the sequence 3′ GTCC 5′ (see the schematics in Figure 3b), where PrimPol can form a *_3p_A*GG trimer by providing [γ-^32^P]*ATP* and dGTP (or ddGTP) as previously described. As shown in Figure 3b (lanes 2–6), PrimPol promptly generated *_3p_A*G dimers and, after a first translocation, *_3p_A*GG trimers; moreover, longer products of 4–5 nt were also produced by reiterative insertions of dG by PrimPol, due to abnormal translocation (via slippage) of the incipient primer strand (generically named “slippage” products). In order to analyse the discrimination against CTNAs when PrimPol is making trimers, two different concentrations of ddGTP (10 and 200 μM) and increasing doses of dGTP were used (Figure 3b, lanes 7–16). At the lowest dose of ddGTP (10 µM), *_3p_A*GddG trimers were clearly observed even in the presence of the same concentration (10 µM) of dGTP (dd/d ratio = 1; Figure 3b, lane 9). At the highest dose of ddGTP (200 µM), a global inhibition in primer synthesis was observed, but even the most favourable ddGTP/dGTP ratio (200-fold) used in this assay was insufficient to detect *_3p_A*ddG dimer synthesis, as described previously (Figure 3a, lanes 14–16); conversely, insertion of ddG did occur at the 3′ end of dimers to form the *_3p_A*GddG trimer, even at a dd/d ratio = 2 (Figure 2b, lane 16), but not at the 3′ end of trimers or larger products such as *_3p_A*GGG, where the primer requires slippage for further extension. The quantification of the dNTP/ddNTP discrimination to form a trimer, calculated as indicated in the Materials and Methods, was 9-fold.

Then, we added an extra dC at the template priming site (3′ GTCCC 5′; see the schematics in Figure 3c) to investigate whether ddGTP can be inserted at the fourth position of the primer when slippage is not yet occurring. Thus, this new template variant facilitates the canonical synthesis of a template-directed tetramer after two conventional translocation events, just by providing [γ-^32^P]*ATP* and dGTP (or ddGTP). Again, ddG could not be observed at dimers or at the end of slippage products (in this case in products longer than 4 mer), but it was incorporated to form trimers (*_3p_A*GddG; lanes 7–16) and to a lesser extent at tetramers (*_3p_A*GGddG; lanes 8–11 and 14–16). ddG-ended tetramers were observed even at dd/d ratios as low as 2 (lane 11) and 4 (lane 16). Quantification of the dNTP/ddNTP discrimination to form a tetramer, calculated as indicated in the Materials and Methods, was 40-fold.

To corroborate that d/dd discrimination is strongly reduced when PrimPol is forming a trimer or a tetramer, we tested the incorporation of another nucleotide distinct from ddG. For this purpose, we used the template sequence 3′-GTCAGACAGCA5′ (with the preferred priming site in bold) flanked by dT tails, which allows a more detailed and sequential analysis of the elongation steps of primer synthesis by PrimPol (see the schematics in Figure 4a,b). Thus, by supplying the reactions with *ATP* as the initiating 5′-ribonucleotide and dGTP as the firstly required 3′ deoxynucleotide, synthesis of *_3p_A*G dimers was promoted (Figure 1a, lane 2); after dimer formation, we could observe that ddTTP was readily incorporated to form the *_3p_A*GddT trimer from the lowest concentration (10 µM) tested (lane 3). Quantification of the dNTP/ddNTP discrimination to extend a *_3p_A*G dimer with either dTTP or ddTTP (calculated from lanes 8 and 9), as indicated in the Materials and Methods, was about 20-fold. In addition to that, when further elongation of the primer was allowed by providing also dCTP and dTTP (lanes 6–9), only a 25-fold excess of ddTTP over dTTP was required to observe a significant block in elongation due to the abortive synthesis of *_3p_A*GddT (lane 9). Therefore, this result reinforces the observation that there is low d/dd discrimination at the third position of the incipient primer. As shown in the schematics (Figure 4b), by supplying the reactions with *ATP* as the initiating 5′-ribonucleotide and the two first 3′ deoxynucleotides (dGTP and dTTP), synthesis of dimers (*_3p_A*G) and trimers (*_3p_A*GT) was promoted (lane 2); then, when ddCTP was additionally provided, *_3p_A*GTddC tetramers were detected from 10 µM ddCTP (lanes 3–6). When dCTP was added together with *ATP*, dGTP, and dTTP, further primer elongation products are allowed (lane 7). Now we can check dCTP/ddCTP discrimination by PrimPol when forming a tetramer. As shown in the gel (lanes 8–11), just a 5-fold higher concentration of ddCTP over dCTP (lane 9) provoked the synthesis of a ddC-blocked 4-mer primer (*_3p_A*GTddC) and the subsequent reduction in the fully elongated primers. The quantification of the dNTP/ddNTP discrimination to extend a *_3p_A*GT trimer with either dCTP or ddCTP (calculated from lanes 9 and 10), as indicated in the Materials and Methods, was about 13-fold.

Taken together, our results show that discrimination against ddNTPs by human PrimPol is especially strong (1400-fold) during the first event of dimer synthesis (Figure 3a–c), suggesting that a 3′-hydroxyl group favours stabilization (and perhaps proper orientation) of the 3′ nucleotide bound at the elongation site, necessary when forming the pre-ternary complex that precedes dimer formation [16]. Conversely, trimer formation is the most susceptible step to incorporate a ddNTP during primer synthesis, having a d/dd discrimination ratio of only 9-fold. Discrimination against a ddNTP is slightly increased during tetramer formation (13-fold), allowing maturation of the nascent primer at moderate concentrations of ddNTPs, to be elongated by replicative polymerases.

### 3.4. Human PrimPol Residue Arg^291^ Is Crucial for dNTP Binding and ddNTP Discrimination

Analysis of the crystal structure of human PrimPol [18] indicates that the triphosphate moiety of the 3′ incoming nucleotide is attached by Lys^165^, Ser^167^, and His^169^ from motif B, Arg^291^, and Lys^297^, all of them belonging to the ModC subdomain which includes also the catalytic residues Asp^114^, Asp^116^, and Glu^280^. As shown in Figure 5a, the side chain of Arg^291^ makes direct interactions with β and γ phosphates of the 3′ incoming nucleotide that might be crucial for substrate orientation and catalysis of the phosphodiester bond. In addition, the main-chain amide of Arg^291^ makes a hydrogen bond with the 3′-OH group of the 3′-incoming dNTP that could contribute to the preferred binding and stabilization of dNTPs at PrimPol’s active centre (see Figure 5a). Therefore, Arg^291^ is a likely candidate to be involved in the discrimination against ddNTPs.

To study the importance of Arg^291^ on the stability of 3′ incoming nucleotides, the arginine was initially changed to a non-conservative alanine (R291A). In order to evaluate the importance of Arg^291^ for the interaction of PrimPol with the incoming nucleotide, we used electrophoretic mobility shift assays (EMSA) to test the capacity of the R291A mutant to form a binary complex with the 3′ incoming nucleotide (PrimPol:dGTP), and a pre-ternary complex with both 3′-nucleotide and ssDNA (PrimPol:ssDNA:dGTP), as recently described [16,17,24] (see the schematics at Figure 5b). Figure 5b shows that elimination of Arg^291^ impedes the formation of both binary and pre-ternary complexes, demonstrating its crucial role in dNTP binding at the elongation site, as inferred from the crystal structure.

To initiate primer synthesis, PrimPol requires a stable interaction with the ssDNA template, and also the contribution of specific residues to stabilize the two incoming dNTPs that will form the initial dimer. The Zn-finger C-terminal domain of PrimPol stabilizes the triphosphate moiety of the 5′-nucleotide, being irrelevant for the binding of the 3′ site nucleotide [16]. As expected, the incapacity of mutant R291A to bind the 3′ dNTP, as inferred from the EMSA assays, resulted in a primase-dead protein (Figure 5d), unable to form initiating *_3p_A*G (lanes 8–10) or *_3p_Add*G (lanes 11–13) dimers. Moreover, semi-conservative mutation of Arg^291^ to Lys (Figure 5d) produced identical results, reinforcing the importance of this invariant arginine for the productive binding of the 3′ incoming dNTP. In agreement with their overall structural integrity, the two mutants R291A and R291K were shown to be as efficient as the wild-type PrimPol to form a stable PrimPol ssDNA binary complex (Figure 5c).

Additionally, as expected, mutant R291A failed to elongate a synthetic primer in a conventional DNA polymerase assay in the presence of the four dNTPs (Figure 5e). Only a small amount of the +1 elongated product (not requiring primer translocation) could be observed from 10 µM dNTPs (lanes 7–9). The more conservative R291K rendered a less dramatic phenotype in DNA polymerization assays, as the primer could be extended by a few nucleotides (Figure 5e, lanes 10–13). Analysis of the kinetic parameters of dNTP insertion in (+1) DNA incorporation assays (Table 2) demonstrated that the catalytic efficiency of these mutants was significantly reduced, only maintaining 1.25% (R291K) and 0.29 % (R291A) of the WT polymerization capacity. Interestingly, the conservative change of Arg^291^ into lysine (R291K) produced a large drop in the affinity for the 3′ incoming dNTPs, as implied from its 54-fold higher K_m_ relative to WT PrimPol (Table 2); conversely, that mutation appears to preserve the proper orientation of the α phosphate, as reflected by the small decrease in the catalytic constant. On the other hand, the non-conservative R291A mutant PrimPol, that has almost a catalytically dead phenotype (Figure 5d,e), showed a 19-fold increase in K_m_ compared to the WT, indicating a loss of affinity for the nucleotide, combined with a similar reduction in the catalytic constant, k_cat_, likely due to an incorrect orientation of the incoming 3′dNTP in the active site of PrimPol, which compromises the nucleophilic attack on its α-phosphorus (Table 2).

In summary, both mutants R291K and R291A showed a great reduction in their primase and polymerase activities in the presence of dNTPs, and a complete inability to use ddNTPs as substrates for catalysis. Collectively, our analysis demonstrates the importance of Arg^291^ for stable binding and suggests a role in the correct positioning of dNTPs at the active centre of human PrimPol.

## 4. Discussion

Replication-blocking agents, such as dideoxynucleotides (ddNTPs), can be hazardous to the very sensitive mitochondrial replicative polymerase, Polγ, which can incorporate ddCTP almost indiscriminately during the replication of the mitochondrial genome [25,26]. In the present work, we questioned how the PrimPol present in mitochondria [7] assists Polγ to cope with this blocking agent. In 2017, Torregrosa-Muñumer et al. [11] demonstrated that PrimPol can reinitiate ddC- and UV-stalled mtDNA replication by priming mtDNA replication from non-conventional origins, mirroring the repriming function of PrimPol in the nucleus [10,12,27]. Whereas PrimPol was required during mtDNA replication under stress conditions, no apparent differences were detected in mitochondrial replication intermediates for both unstressed WT or PrimPol^−/−^ MEF cells. That showed that PrimPol is not essential for unchallenged mtDNA replication [11], in agreement with its dispensable nuclear role in unstressed cells [10]. However, treatment with ddC causes accumulation of mitochondrial replication intermediates only in PrimPol-competent cells, due to recurrent initiation and stalling events [11]. In the absence of PrimPol, no re-initiation occurs, resulting in the loss of replication intermediates, further demonstrating that PrimPol is required for replication maintenance of mtDNA in the presence of ddC.

Here, we demonstrate that PrimPol strongly discriminates (325- to >700-fold) against the four ddNTPs when performing DNA polymerization in vitro in the presence of manganese ions (Table 1), an activating metal ion which is considered to be physiological for human PrimPol [7,24,28]. These results are in good accordance with Mislak and Anderson [15], who firstly showed that PrimPol discriminates against ddATP and ddCTP better than Polγ during polymerization, implying low PrimPol-mediated mitochondrial toxicity with these analogues. We early showed that PrimPol has a great capacity to bypass 8-oxoG during primer extension, either by selecting the correct dC or, alternatively, the promutagenic dATP insertion opposite this lesion [7]. Here, we show that PrimPol keeps a strong discrimination against ddCTP, and even stronger against ddATP, when copying an 8-oxoG template lesion (Table 1). The oxidized base 8-oxoG is believed to slow down the mitochondrial replisome due to the poor translesion capacity of the mitochondrial replicative machinery [29]. However, according to Stojkovič et al. [29], PrimPol does not alleviate pausing sites by acting as a canonical TLS polymerase, since probably it cannot displace the replicative polymerase Polγ.

Otherwise, PrimPol could assist 8oxoG-induced fork stalling by generating a new primer beyond the 8-oxoG lesion, that will be further elongated by Polγ, to resume mtDNA synthesis. Therefore, it was relevant to evaluate if the primase activity of PrimPol is affected by the presence of ddNTPs, paying special attention to the sequential events starting from the synthesis of a dimer, to becoming a mature DNA primer. Our results demonstrate that human PrimPol can synthesize mature primers in the presence of chain-terminating ddNTPs, taking advantage of a moderate discrimination against ddNTPs which favours binding and insertion of dNTPs at the PrimPol active site. This discrimination against ddNTPs is maximized during dimer formation (1400-fold), a rate-limiting step that requires a stable binding of a complementary 3′ nucleotide in a pre-ternary complex with ssDNA, and a subsequent and adjacent binding of a complementary 5′dNTP (or 5′NTP) mediated by the Zn-finger domain of PrimPol [16]. The large d/dd discrimination ratio calculated here for dimer formation on defined ssDNA templates (1400-fold) is compatible with the low ddG inhibition observed when dimers were allowed to be formed on M13ssDNA [11]. Strikingly, the first elongation step after dimer formation, perhaps reflecting a conformational change in the Zn-finger domain to accommodate the first translocation event, has a largely reduced discrimination against ddNTPs (9-fold), allowing an easy formation of ddN-terminated trimers (an Achilles’s heel vulnerability). Further elongation events are expected to recover higher dN/ddN discrimination values, which allow maturation of the incipient primer in the presence of ddNTPs.

To investigate how PrimPol discriminates between natural dNTPs and analogue ddNTPs, we took advantage of the 3D structure of the catalytic core of human PrimPol, in a ternary complex with DNA and dATP [23]. The crystal structure identified several residues as ligands of the phosphate groups of the 3′ incoming nucleotide (Lys^165^, Ser^167^, His^169^, Arg^291^ and Lys^297^), but only one of them (Arg^291^) interacts with the 3′-OH group of the incoming nucleotide, which is absent in ddNTPs (see Figure 5a). Substitution of Arg^291^ for Lys or Ala resulted in a strong loss of polymerase activity (Figure 5d) mainly due to a decreased affinity for 3′ incoming nucleotides, reflected by the elevated K_m_ in both mutants (Table 2), and a much lower k_cat_ in R291A. On top of that, R291K and R291A completely lost their primase activity (Figure 5c,d), again as a consequence of a reduced 3′ dNTP binding capacity that impedes formation of a pre-ternary complex (ssDNA:PrimPol:dGTP; Figure 5b), a prerequisite to form the initiating dimer. Our estimation that PrimPol has a lower affinity for ddNTPs (K_m_ is up to 72-fold higher for ddNTPs than for dNTPs) and a decreased catalytic constant between 1 and 2 orders of magnitude suggests that the multiple interaction of Arg^291^ with the β and γ phosphates of the incoming 3′ nucleotide, but also with its 3′-OH group, is likely crucial to determine nucleotide orientation and subsequently for stable binding of dNTPs and efficient catalysis.

Insertion of a CTNA at the mitochondrial fork is unlikely to happen under normal physiological conditions, unless in patients undergoing treatment where a CTNA is prescribed. ddC is not used anymore in many countries as CTNA therapy due to its high mitochondrial toxicity [26]; however, ddC has shown to be an interesting tool to study mitochondrial DNA replication, since Polγ can easily incorporate ddCTP [(k_cat_/K_m_)_dCTP_/(k_cat_/K_m_)_ddCTP_ = 7] [30], causing fork stalling or mtDNA depletion. Although PrimPol function can restore the progression of the replication fork in the presence of ddCTP, recurrent insertion of this analogue by Pol**γ** can leave behind many gaps in the newly synthesized mtDNA. Contrary to the efficient incorporation of ddCTP by Polγ, removal of this CTNA by its proofreading activity is a very slow and inefficient reaction, explaining the persistence of ddCMP in mtDNA, and the toxicity of CTNAs [5,25]. Hence, in a situation where Polγ has stalled the mtDNA replication by inserting ddCTP or encountering several 8-oxoG base modifications in the template, PrimPol can potentially rescue the mitochondrial replication by making a new primer ahead of the stalled replicative machinery, avoiding the ddCTP insertion due to the discrimination role of Arg^291^ stabilization of dNTPs at the active site of PrimPol. However, this rescue does not completely recover mitochondrial DNA replication, resulting in the already known mitochondrial toxicity of ddCTP.

## 5. Conclusions

In this work, we have determined the high discrimination of PrimPol against ddNTPs during polymerization and primer synthesis, especially when forming the initial dimer. PrimPol Arg^291^ is identified here as a key ligand for the stabilization and proper orientation of 3′ incoming deoxynucleotides. A direct interaction of Arg^291^ with the 3′-OH group of the dNTP is likely required to orient their β and γ phosphates for binding (also by Arg^291^), optimizing both maximal stability and the right positioning for catalysis. That would explain the low affinity for ddNTPs, thus preventing their incorporation. This PrimPol selectivity for natural dNTPs suggests a protective role of PrimPol during mitochondrial DNA replication, when repriming is needed to rescue replication forks stalled by ddCTP or other CTNAs inserted by Polγ. However, this damage mitigation by PrimPol is not enough to avoid ddCTP mitochondrial toxicity, as clinical pharmacology has proven.

## Figures and Tables

**Table 1:** Kinetic parameters for dNTP or ddNTP incorporation opposite the complementary template base (G, C, T, A, and 8oxoG).

Template	Nucleotide	K_m_ (µM)	k_cat_ (s^−1^)	k_cat_/K_m_ (s^−1^ · µM^−1^)	Discrimination (dNTP/ddNTP) ^a^
G	dCTP	0.82	1.88 × 10^−2^	2.29 × 10^−2^	330
	ddCTP	36.43	2.53 × 10^−3^	6.95 × 10^−5^	
C	dGTP	0.48	8.43 × 10^−3^	1.74 × 10^−2^	391
	ddGTP	23.52	1.04 × 10^−3^	4.44 × 10^−5^	
T	dATP	0.49	9.69 × 10^−3^	1.98 × 10^−2^	719
	ddATP	35.37	9.73 × 10^−4^	2.75 × 10^−5^	
A	dTTP	0.84	1.28 × 10^−2^	1.53 × 10^−2^	325
	ddTTP	13.24	6.23 × 10^−4^	4.70 × 10^−5^	
8oxoG	dCTP	0.78	1.50 × 10^−2^	1.93 × 10^−2^	345
	ddCTP	26.54	1.48 × 10^−3^	5.59 × 10^−5^	
	dATP	1.18	1.48 × 10^−2^	1.26 × 10^−2^	n.d.
	ddATP	n.d.	n.d.	n.d.	

^a^ Calculated as [(k_cat_/K_m_)_dNTP_/(k_cat_/K_m_)_ddNTP_]. n.d. Non-determined as no incorporation of ddATP opposite a 8-oxoG was detected using our methods.

**Table 2:** Kinetic parameters for dNTP insertion by WT *Hs*PrimPol and Arg^291^ mutants.

Protein	K_m_ (µM)	k_cat_ (s^−1^)	k_cat_/K_m_ (s^−1^ · µM^−1^)	Relative Activity ^a^
WT	0.82	1.88 × 10^−2^	2.29 × 10^−2^	100%
R291K	44.46	1.26 × 10^−2^	2.86 × 10^−4^	1.25%
R291A	15.5	1.05 × 10^−3^	6.75 × 10^−5^	0.29%

^a^ Calculated as [(k_cat_/K_m_)Protein/(k_cat_/K_m_)WT]·100.

## Data Availability

MDPI Research Data Policies.
